# Application of Small Epigenetic Modulators in Pediatric Medulloblastoma

**DOI:** 10.3389/fped.2018.00370

**Published:** 2018-12-03

**Authors:** Clemens Zwergel, Annalisa Romanelli, Giulia Stazi, Zein Mersini Besharat, Giuseppina Catanzaro, Marco Tafani, Sergio Valente, Antonello Mai

**Affiliations:** ^1^Department of Drug Chemistry and Technologies, Sapienza University of Rome, Rome, Italy; ^2^Department of Experimental Medicine, Sapienza University of Rome, Rome, Italy; ^3^Istituto Pasteur-Fondazione Cenci Bolognetti, Sapienza University of Rome, Rome, Italy

**Keywords:** brain cancer, medulloblastoma, epigenetic modulators, targeted therapy, innovative therapy concepts

## Abstract

Medulloblastoma is one of the most frequent among pediatric brain tumors, and it has been classified in various subgroups. Some of them already benefit from quite good therapeutic options, whereas others urgently need novel therapeutic approaches. Epigenetic modulators have long been studied in various types of cancer. Within this review, we summarize the main preclinical studies regarding epigenetic targets (such as HDAC, SIRT, BET, EZH2, G9a, LSD1, and DNMT) inhibitors in medulloblastoma. Furthermore, we shed light on the increasing number of applications of drug combinations as well as hybrid compounds involving epigenetic mechanisms. Nevertheless, in the studies published so far, mainly un-specific or old modulators have been used, and the PKs (brain permeability) have not been well-evaluated. Thus, these findings should be considered as a starting point for further improvement and not as a final result.

## Introduction

Medulloblastoma (MB) is one of the most frequent and extensively studied pediatric brain tumors. According to the WHO-classification of central nervous system tumors, four main genetically defined subgroups have been described: WNT, SHH, group 3, and group 4. Each of these groups has its unique expression signature and clinical outcome (1–4). Guerreiro Stucklin et al. recently well-summarized the differences in biological and clinical behavior between subgroups (3). Because of the heterogeneity of the various groups of MB, a targeted and efficient therapy, specifically for young patients, is very challenging (2). Epigenetic modulators have long been studied in various types of cancers, and some of them have been approved mainly for the treatment of hematological malignancies (5). These compounds are a particularly appealing therapy approach because they do not alter irreversibly the genetic code but act on reversible epigenetic marks, with a lower risk of side effects. In MB, a malignant brain tumor, the main challenge relies on the fact that whatever small molecule used as a therapeutic agent has to be able to cross the blood-brain barrier (6). The molecular epigenetic deregulation in MB has been recently reviewed, shedding light onto the pathways involved in the disease, on their biological importance as well as on the possible targets to hit (7, 8).

In this review we would like to highlight the latest preclinical and clinical efforts regarding the application of epigenetic modulators in MB. An overview of the presented compounds, their targets and effects in MB can be found in Table 1.

**Table 1:** Summary of the epigenetic modulators and combinations active in MB.

**Compound**	**Structure**	**Target**	**Results**	**Combination**
Suberoylanilide-hydroxamic acid, vorinostat	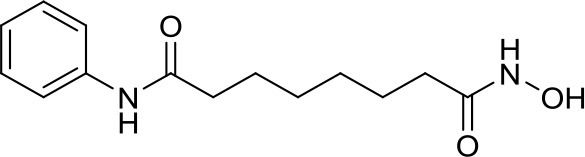	HDACs	Active in DAOY and D283med MB cancer stem cells (13) Active in HD-MB03 cell line and xenograft model (15) Efficiently reduced the metabolic activity in MYC-MB cells (10).	Synergistic effect in D283med cells, but not in DAOY with decitabine (41). Newer study for both cell lines (13). Synergistic effects of VPA and vorinostat with irradiation in MB (42). The aurora kinase inhibitor MLN8237 had additive inhibition effects on MB group 3 cell lines (43)
Romidepsin	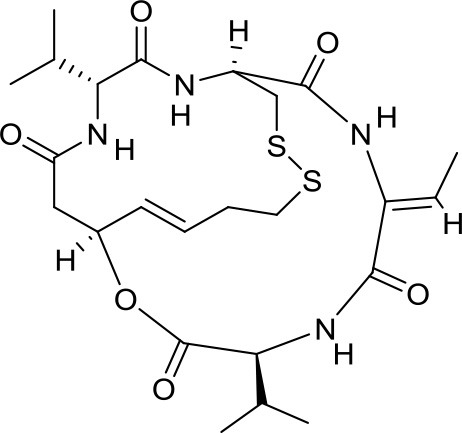	HDAC1/2	Active in DAOY and D283med MB cancer stem cells (13)	–
Panobinostat	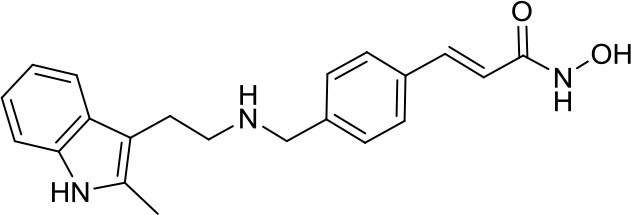	HDACs	Suppressed leptomenigeal seeing in a MB mouse model (14) Active in HD-MB03 cell line and xenograft model (15)	–
Valproic acid, VPA	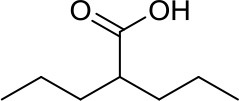	Class I/IIa HDACs	Potential treatment in various MB cell lines (11, 12)	Synergistic effects of VPA and vorinostat with irradiation in MB (42)
Parthenolide	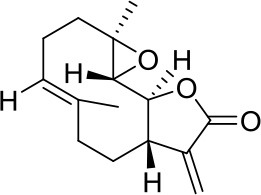	HDAC1	Active in DAOY and D283med MB cancer stem cells (13)	–
MS-275, entinostat	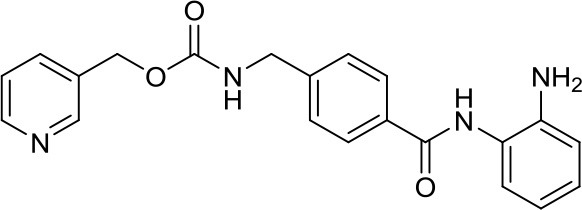	Class I HDACs	Active in DAOY and D283med MB cancer stem cells (13) Efficiently reduced the metabolic activity in MYC-MB cells. (10)	–
HDiA	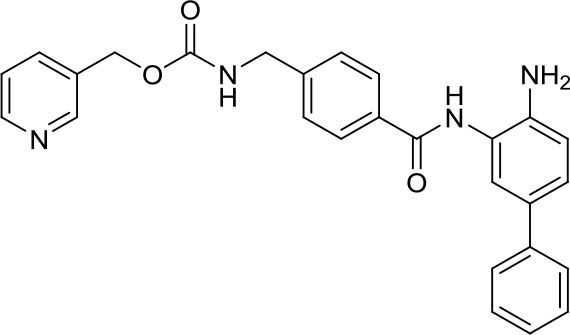	HDAC1/2	Block GLI1/2 activities and SHH MB growth (16)	–
HDiB	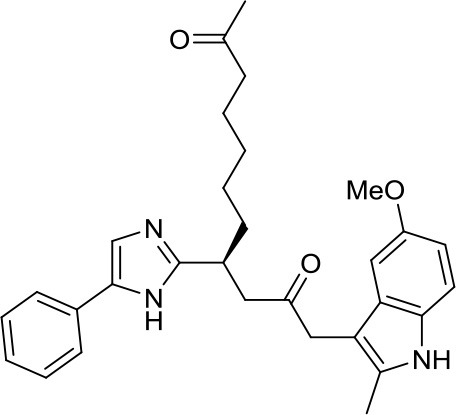	HDAC1/2	Block GLI1/2 activities and SHH MB growth (16)	–
Curcurmin	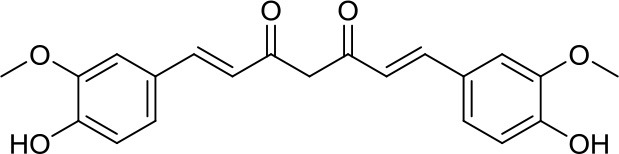	HDACs	Increased survival in the Smo/Smo transgenic MB mouse model (17)	–
MAZ1863	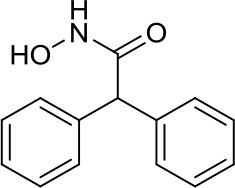	Class IIa HDACs	Only very weak effects on MYC-MB cells (10)	–
MAZ1866	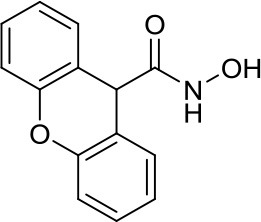	Class IIa HDACs	Only very weak effects on MYC-MB cells (10)	–
TH34	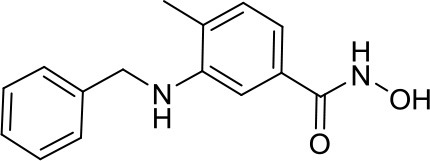	HDAC 6/8/10	Induced caspase-dependent programmed cell death in various MB cell lines (18)	–
Nicotinamide	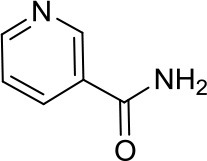	SIRTs	SIRT1 inhibition might be a double edge sword in MB treatment (24)	–
JQ-1	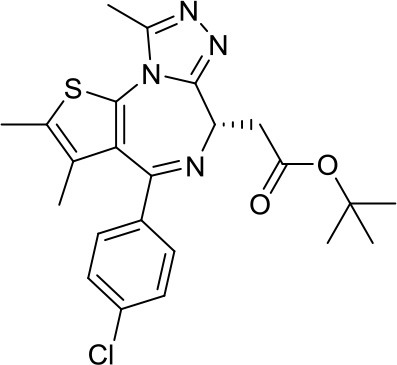	BETs	Decreased proliferation and tumor growth in SHH MB via reducing the expression of. GLI1 and GLI2 (27) Active in a human group 3 MB xenograft model via MYC downregulation (28–30)	Effective combination with the CDK inhibitor milciclib, as both regulate the MYC function in MB via different actions, prolonging survival in a MB animal model (32)
I-BET151	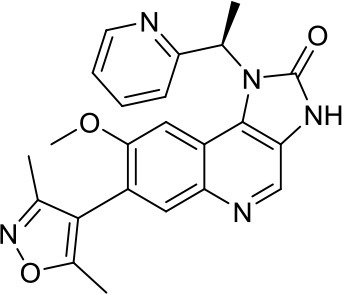	BETs	Inhibition of the SHH pathway in SHH-MB cells as well as in a MB mouse model (31)	–
Decitabine	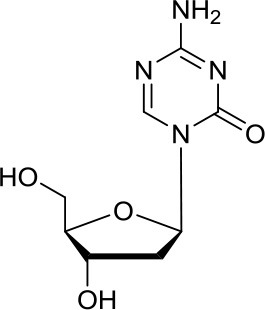	DNMTs	Found to be quite inactive in DAOY (13, 38) and UW228 MB cells (38) as well, differently D283med cells were quite sensitive (13)	Synergistic effect in D283med cells, but not in DAOY with decitabine (41). Newer study for both cell lines (13). Triple combination of decitabine/irradiation and abacavir turned out to work effectively in various MB cell lines (41) Phenylbutyrate in combination with decitabine and the tyrosine kinase inhibitor Gleevec induced apoptosis in DAOY and UW228 3 MB cell lines (38).
Zebularine	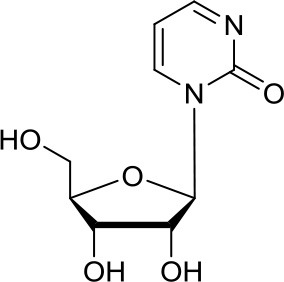	DNMTs	Inhibits the expression of SHH pathway components, such as SMO and GLI1, in DAOY and ONS-76 MB (39)	DNMTi zebularine has been tested in combination with vincristine in SHH MB cells, displaying a synergistic effect (39)
MC2840 (compound 2)	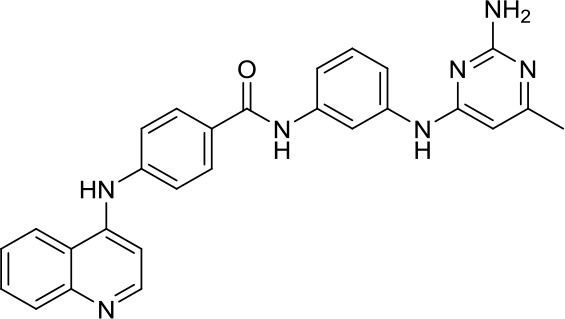	DNMTs	Impaired MB-SC growth led to high MB-SC differentiation rates (40)	–
MC3343 (compound 5)	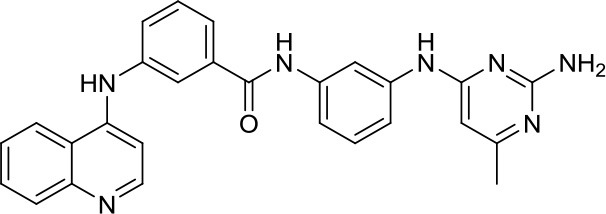	DNMTs	Significantly impaired the MB-SC growth rate (40)	–
3-Deazaneplanocin A, DZNep	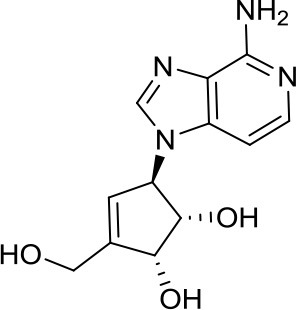	EZH2	Indirect and rather unspecific EZH2i in MB (33)	–
MC3629	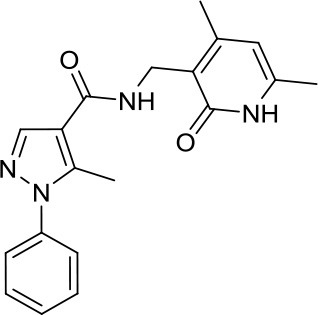	EZH2	Reduces in a MB xenografted mice the tumor volume, stemness and cell proliferation and lastly induces apoptosis (34)	–
UNC0638	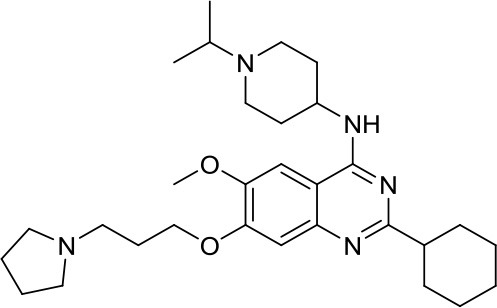	G9a	Reduces DAOY proliferation via controlling the USP37 expression mediated by G9a (35)	–
SP2509	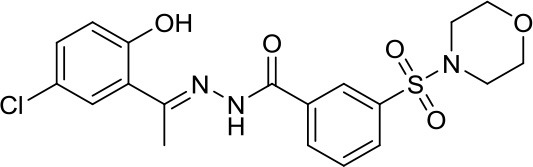	LSD1	Disruptor of the CoREST–LSD1 complex active in various MB cell lines (36)	–
4SC-202	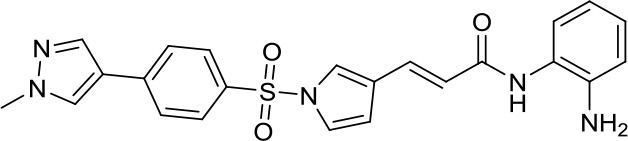	HDAC1/2/3 and LSD1	–	Active in various MB cell lines (36)
Sodium Phenylbutyrate	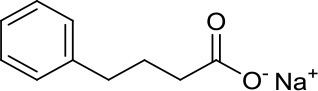	HDACs	–	Phenylbutyrate in combination with decitabine and the tyrosine kinase inhibitor imatinib induced apoptosis in DAOY and UW228 3 MB cell lines (38)
NL-103	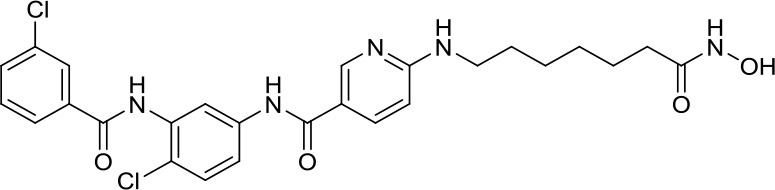	HDAC/HH	–	NL-103 is a dual inhibitor of the HDACs and HH pathway with potential activity in MB (44)

## Single epigenetic modulators in medulloblastoma

### Histone (De)acetylation modifiers/readers

#### HDAC inhibitors (HDACi)

HDACi are among the oldest and deeply studied class of epigenetic modulators. In time, the most widely studied HDACi have not been isoform selective, but they were targeting more than one HDAC, specially class I and/or IIa/b HDACs. Nevertheless, selective isoform-specific modulators are more and more developed (9). Vorinostat, romidepsin, and belinostat are FDA-approved drugs to treat rare T-cell lymphomas by re-expressing silenced tumor suppressor genes. Currently, many preclinical studies are evaluating the effects of these inhibitors on MB (8). Surprisingly, relatively few clinical studies have been conducted with these validated inhibitors in MB, and most of them are closed or finished (10).

Older studies described valproic acid (VPA) as a potential treatment in various MB cell lines (11, 12), nevertheless this compound seems no longer under evaluation as newer studies cannot be found. Parthenolide, an HDAC1i, has been shown to be active in DAOY and D283med MB cancer stem cells, which aberrantly overexpress HDAC1 (13). In the same study also other HDACi, such as vorinostat, entinostat, or romidepsin, were tested resulting less active. However, the authors of this study noticed that very low concentrations of the HDACi resulted in an increase, rather than a decrease, in proliferative activity (13).

Recently, another well-known pan-HDACi, panobinostat, was reported to suppress leptomeningeal seeding, a rare complication in MB spreading, causing brain and spinal cord inflammation in a mouse model (14).

Milde et al. developed a Group 3 MB HD-MB03 cell line and xenograft model with high HDAC expression levels and sensitivity to HDACi, such as vorinostat and panobinostat (15).

A meaningful example of the involvement of HDACi in the SHH signaling pathway has been given by Canettieri et al. They showed that the HDAC1/2 selective inhibitors HDiA and HDiB blocked GLI1 and GLI2 activity through their acetylation, and SHH MB cell growth in several SHH MB cell lines (16). Despite these interesting results, no follow up studies have been published so far.

The natural compound curcumin, through HDAC inhibition and HDAC4 level depletion, reduced tumor growth and significantly increased survival in the Smo/Smo transgenic MB mouse model displaying HDAC4 overexpression. However, due to the pleiotropism displayed by curcumin, these positive results might not only be ascribed to HDAC4 inhibition but also to other off-target effects (17).

In 2015, Ecker et al. used the class IIa-selective HDACi MAZ1863 and MAZ1866 in Group 3 MB cancer cells and compared them to vorinostat (pan-HDACi) and to the class I specific inhibitor MS-275 (entinostat). MAZ1863 and MAZ1866 had only very weak effects on MYC-MB cells, whereas vorinostat and entinostat efficiently reduced the metabolic activity in MYC-MB cells. These results give precious hints on the development of novel therapies with selective HDACi in MYC- dependent MB (10).

Interestingly, when tested in the MED8A MB cell line, the novel non-toxic HDAC6/8/10 inhibitor TH34 modestly impaired colony growth and specifically induced caspase-dependent programmed cell death in a dose-dependent manner (18). TH34 warrants deeper evaluation and could be an interesting candidate for *in vivo* studies.

To sum up, the well-established and approved HDACi have so far failed to demonstrate a significant antitumoral effect in solid malignancies in preclinical and clinical settings (19), in contrast to leukemias and lymphomas. The failure of translating preclinical results into clinical success has been extensively discussed (20). Most likely, insufficient pharmacological study design regarding the clinical situation such as compound concentrations and their pharmacokinetic as well as dynamic properties are the primarily suspected factors (21).

#### SIRT inhibitors

Sirtuins (SIRTs), also known as class III HDACs, are NAD^+^ dependent deacetylases considered as a separate family of enzymes including seven different isoforms (hSIRT1-7). So far, there is very little literature evidence about the use of SIRT modulators in MB (22). In 2013, Ma et al. demonstrated that SIRT1 was overexpressed in human MB cells. In their work, they showed that lowered SIRT1 expression levels by siRNA or SIRT1 pharmacological inhibition with nicotinamide resulted in growth arrest and apoptosis in MB cells (23).

In contrast, Tiberi and coworkers found that the downregulation of the BLC6/BCOR/SIRT1 complex, a potent repressor of the SHH pathway, led to MB growth in human cells and in a mouse model. They demonstrated that SIRT1 is necessary for the BCL6 function (24), thus SIRT1 inhibition might be a double-edged sword in MB treatment. Therefore, researchers should proceed with caution for SIRT1 modulation in MB. The different results reported by these research groups well-summarize the problem of the context-dependent function of epigenetic targets (in this case SIRT1) in different experimental settings and MB subgroups.

#### BET inhibitors (BETi)

The BET (Bromodomain and Extra-Terminal domain) proteins BRD2, BRD3, and BRD4, have been extensively studied in brain tumors including MB (25). These proteins are epigenetic readers as they recognize acetyl-lysine residues and acetylated chromatin, which usually mark active enhancers, thus they are important mediators of gene activation. High levels of H3K27Ac mark super-enhancers regulate key genes in cancer growth, and are sensitive to BET inhibition (26).

The BETi JQ-1 is one of the most studied in the literature. Tang et al. demonstrated that reduced expression of BRD4 via RNAi or its pharmacologic inhibition by JQ-1 resulted in decreased proliferation and tumor growth in SHH MB, reducing the expression of the glioma-associated oncogenes GLI1 and GLI2 (27). The same compound also led to positive results in Group 3 MB, as MYC-driven MBs are sensitive to BETi. Henssen et al. described JQ-1 to be active in a human Group 3 MB xenograft model via MYC downregulation, as it reduced tumor volume and prolonged survival rates (28). Similar results, corroborating the potential of JQ-1 in downregulating MYC expression, have been obtained by two other independent research groups (29, 30). Furthermore, JQ-1 has been demonstrated to block stem cell-associated signaling and was able to induce cell senescence in a MYC-MB cellular model as well as in xenograft mice (30).

Another BETi, namely I-BET151, has been shown to provide biological effects similar to JQ-1 in SHH MB. More precisely, this compound reduced the BRD4 binding to the GLI1 gene locus, thus resulting in the inhibition of the SHH pathway in SHH MB cells as well as in a MB mouse model (31).

Currently, JQ-1 is not in clinical trials for MB treatment due to its poor pharmacokinetic and pharmacodynamic properties (32). It is quite surprising that other BETi similar to JQ-1, such as RG6146 (TEN-010) or OTX105, which are currently evaluated in clinical trials for other cancer types [ClinicalTrials.gov NCT01987362, NCT02259114], have never been tested in MB even in preclinical studies. Nevertheless, BETi represent a promising strategy to follow the development of novel MB therapies.

### Histone (De)methylation modifiers

#### EZH2 inhibitors (EZH2i)

Enhancer of zeste homolog 2 (EZH2) is a histone lysine *N*-methyltransferase involved in the PRC2 (Polycomb Repressive Complex 2), which has been widely studied in cancer including MB. One of the first published studies on MB used the rather toxic DZNep, an inhibitor of S-adenosyl-L-homocysteine hydrolase, as an indirect and quite unspecific EZH2i (33). Recently, our research group published the pyrazole compound MC3629 as a simplified analog of the two different SAM-competitive EZH2i EPZ005687 and GSK2816126. This particular compound was not only active in human SHH MB cancer cell models, where it significantly impaired H3K27me3 and PCNA protein levels leading to apoptosis, detected as an increased level of cleaved caspase 3, but also, to our knowledge for the first time, in a SHH MB murine model. Importantly, MC3629 better penetrated the blood-brain barrier *in vitro* and *in vivo*, when compared to the parent compound GSK2816126. This might explain at least in part why MC3629, despite its lower *in vitro* potency, efficiently reduced H3K27me3 levels in brain and cerebellum of MB xenografted mice leading to decreased tumor volume, reduced stemness and cell proliferation ability, and, lastly, induction of apoptosis (34). These encouraging results confirm the importance of EZH2 in MB.

#### G9a inhibitors

The deubiquitylase USP37 was identified as a target of REST, one of the main regulatory complexes in brain development and neurogenesis with aberrant overexpression in MB (5). Dobson et al. showed that the downregulated USP37 in human MB could be re-expressed after G9a inhibition. In more details, G9a catalyzes mono- and di-methylation of histone H3K9, and its histone methyltransferase activity correlated with gene repression of USP37 in MB. The USP37 promoter in MB possesses a significant level of histone H3K9 trimethylation, which was considerably diminished upon treatment of the DAOY cells with the G9a inhibitor UNC0638. This has been the first and unique pivotal study highlighting the importance of G9a inhibition, leading to arrest of MB cell proliferation via control of the USP37 expression (35). However, this is only the first step toward a G9a-based MB treatment, as this target needs to be further validated not only in other MB models but also in opportune *in vivo* studies.

#### LSD1 inhibitors (LSD1i)

Lysine-specific demethylase 1 (LSD1), also known as KDM1A, has been the first of several protein lysine demethylases to be discovered. The modulation of this enzyme has also been studied in MB. Recently, it has been shown that SP2509 inhibited the enzymatic activity of LSD1 rather than acting as a protein-protein disruptor of the CoREST–LSD1 complex. SP2509 was able to block the growth of various human MB cell lines (DAOY, D283med, and ONS-76) through direct LSD1 inhibition (36). This study has a pivotal role since it could be used as a starting point for deeper mechanistic studies as well as for a novel therapeutic approach in MB.

### DNMT inhibitors (DNMTi)

DNA methyltransferases (DNMTs) are a family of enzymes that catalyze the transfer of a methyl group to the C5-cytosine residue of DNA. Aberration of DNA methylation leads to a wide variety of diseases, including cancer. DNMTi are one of the most studied epigenetic modulators after HDACi in cancer. The nucleoside analogs azacytidine and decitabine have been approved by FDA mainly in hematological malignancies (37). These compounds inhibit DNMTs after being incorporated into the DNA, leading to reduced methylation levels often resulting in enhanced tumor suppressor gene expression and finally in increased apoptosis (37). Decitabine was found to be quite inactive in DAOY (13, 38) and UW228 MB cells (38); differently, D283med cells seemed to be quite sensitive to the treatment with this inhibitor (13). Another nucleoside inhibitor, zebularine, inhibits the expression of SHH pathway components, such as SMO and GLI1 in DAOY and ONS-76 MB cell lines, leading to inhibition of their proliferation and to increase of apoptosis rates (39). The main problems of these nucleoside analogs are their poor chemical stability and high toxicity (37). Relatively few non-nucleoside inhibitors are known to date. One of them, developed in our research group has also been tested in MB. Compound 5 and compound 2, both structural isomer of the SGI-1027, have been tested for the first time as non-nucleoside inhibitors in mouse MB stem cells (MB-SC), expressing high levels of DNMT1. Compound 5 arrested the cell clonogenic activity impairing MB-SC growth rate, evaluated by quantification of PCNA levels, and induced cell adhesion and differentiation, evaluated by βIII-tubulin. In these assays, compound 5 displayed the highest growth arrest, while compound 2 induced higher differentiation already after treatment with lower doses (40). Both compounds are interesting tools for further *in vivo* validation, but also a starting point for further drug development.

## Combinations containing at least one epigenetic modulator and hybrid compounds

Chemoresistance is one of the key reasons why drug combinations are applied in therapy. Targeting a disease by just one active principle often results in drug resistance. This problem might be overcome by using two different drugs that target two different molecular pathways involved in the same disease. In MB, this strategy has also been used to various epigenetic modulators in combination with other molecules either targeting epigenetic pathways or non-epigenetic ones. Furthermore, we shed light on novel, innovative hybrid compounds targeting at least one epigenetic molecule as following.

### Combinations of two (or more) epigenetic modulators

Patties et al. published in a first study the effects of combination of several epigenetic modulators, such as the DNMTi decitabine, VPA and vorinostat as HDACi, in MB and later they extended the previous study with the use of irradiation, a common physical therapy approach to fight various cancers (41).

They discovered that the treatment of D283med cells with vorinostat and decitabine produced a synergistic effect in reducing tumor cell viability, whereas the exposure of DAOY cells to the same compounds did not have a synergistic effect (41). However, a more recent study by Yuan et al. resulted in a synergistic effect in both cell lines (13). This example shows that the precise assay conditions as well as concentrations of the drugs, are crucial for the outcome of a study. The last researchers also tested parthenolide in combination with decitabine obtaining a synergistic effect (13). Despite the numerous evidences of synergism by HDACi and DNMTi co-treatment, the precise mechanism of their interplay still needs to be elucidated (13, 41).

Interestingly, also the combination of VPA or vorinostat with irradiation showed similar effects compared to decitabine/irradiation treatments on the mentioned cell lines, even though to a lower extent (42). The latter most powerful combination deserves *in vivo* validation. Also, the combination experiments without irradiation might provide a promising alternative therapeutic strategy, lowering the possibility of resistance.

### Combinations of epigenetic and non-epigenetic modulators

Patties et al. did not only evaluate the combination of several epigenetic modulators, but also combined them with abacavir, a nucleoside analog HIV reverse transcriptase inhibitor, with or without irradiation (41, 42). Abacavir is not only known as an approved drug for HIV-treatment, but possesses also potent anti-cancer effects due to its ability to inhibit the telomerase activity, often overexpressed in several cancers (41). The triple combination of decitabine, abacavir, and irradiation turned out to work effectively in all three tested cell lines (DAOY, MEB-Med8a, D283med), warranting further *in vivo* investigations (41). Vorinostat has also been tested in association with the aurora kinase inhibitor MLN8237, leading to proliferation arrest in Group 3 MB cell lines (43).

Marino et al. evaluated the pan-HDAC inhibitor 4-phenylbutyrate in combination with the DNMTi decitabine and the tyrosine kinase inhibitor imatinib. The co-treatment reduced global methylation and induced apoptosis in DAOY and UW228 3 MB cell lines (38).

The DNMTi zebularine has been tested in combination with the well-known anti-cancer agent vincristine, able to interact with microtubules and tubulin, in SHH MB cells, displaying a synergistic effect (39). All the aforementioned studies are combining approved drugs or compounds which have already been extensively studied in the preclinical and clinical stage for other malignancies.

However, also newer chemical entity combinations have been tested in MB. As reported earlier, MYC is an important player in Group 3 and Group 4 MB. JQ-1 is influencing this pathway via BET inhibition and has been combined with the CDK inhibitor milciclib, because CDKs regulate events in MYC function as well. This combination was well-tolerated, reduced tumor cell growth, and significantly prolonged survival in MB animal model (32). In the future, the combination between BETi and CDKi could be further evaluated using in combination with milciclib, already in clinical trials [NCT01011439, NCT01301391], a BETi more drug-like than JQ-1.

However, for the treatment of MB all the aforementioned combinations are still in their early stage and need to be carefully evaluated before proceeding to the clinical area.

### Hybrid compounds

Hybrid compounds are single chemical entities hitting more than one target. Some of these innovative compounds have been also tested in MB. Inui et al. evaluated the dual HDAC1/2/3 and LSD1 inhibitor 4SC-202 in various MB cell lines (DAOY, D283med, and ONS-76). This compound proved to be active targeting both enzymes in the CoREST-HDAC-LSD1 complex. This study is one of the first examples using dual epigenetic inhibitors in MB (36). 4SC-202 deserves a deeper study regarding its detailed mechanism of action as well as further evaluation as a novel innovative therapy weapon.

NL-103, a dual inhibitor of the HDACs and SHH pathway, shows a hybrid structure merging those of vismodegib, a smoothened receptor (SMO) inhibitor approved by FDA for other solid cancers, and vorinostat, which is known to target the SHH pathway by influencing the acetylation status of GLI1 and GLI2 (16). This innovative compound, with its unique dual-targeted activities, was able to inhibit SHH signaling pathway acting on two different targets, in the oncogene fibroblast model cell line NIH-3T3, where it was more effective than the treatment with single targeting compounds. Thus, in this study such a hybrid was proposed as an attractive candidate to be tested in HH-sensitive MB, however it remains still elusive since no further studies have been published so far (44).

## Conclusions and perspectives

In this review, we have summarized and highlighted epigenetic modulators as promising drug targets in MB. However, there is still a long way to go: mainly not very specific, or older modulators have been used and often the brain permeability has not been well-evaluated. The molecular genetics and detailed epigenetic modulation of the various MB subgroups need to be further studied. As the MB subgroup is a key factor in choosing the right treatment, the development of personalized medicine with highly specific modulators could be a key in improving the poor survival rates of some MB subgroups. Therefore, research should not only focus on the design of more specific and selective epigenetic modulators, but also should study deeper the more biologically oriented factors, such as the tumor molecular genetics, the functional analysis of epigenetic factors and their potential modulation. In both cases, epigenetic modulators can be useful not only as tools to better understand the molecular mechanisms in MB, but also as novel potential drugs for innovative personalized treatments.

## Author contributions

The literature research was conducted by all authors and the manuscript was written thereafter by the contribution of all authors. AM read, corrected, and supervised and coordinated all the work. All contributors read and approved the manuscript.

### Conflict of interest statement

The authors declare that the research was conducted in the absence of any commercial or financial relationships that could be construed as a potential conflict of interest. The handling editor declared a past co-authorship with the authors GC and ZB.

## References

[1] Louis DN Perry A Reifenberger G von Deimling A Figarella-Branger D Cavenee WK. The 2016 world health organization classification of tumors of the central nervous system: a summary. Acta Neuropathol. (2016) 131:803–20. 10.1007/s00401-016-1545-1 27157931

[2] Archer TC Sengupta S Pomeroy SL. Brain cancer genomics and epigenomics. Handb Clin Neurol. (2018) 148:785–97. 10.1016/B978-0-444-64076-5.00050-8 29478614

[3] Guerreiro Stucklin AS Ramaswamy V Daniels C Taylor MD Review of molecular classification and treatment implications of pediatric brain tumors. Curr Opin Pediatr. (2018) 30:3–9. 10.1097/MOP.0000000000000562 29315108

[4] Ivanov DP Coyle B Walker DA Grabowska AM. *In vitro* models of medulloblastoma: choosing the right tool for the job. J Biotechnol. (2016) 236:10–25. 10.1016/j.jbiotec.2016.07.028 27498314

[5] Mazzone R Zwergel C Mai A Valente S. Epi-drugs in combination with immunotherapy: a new avenue to improve anticancer efficacy. Clin Epigenet. (2017) 9:59. 10.1186/s13148-017-0358-y 28572863 PMC5450222

[6] Zhang P de Gooijer MC Buil LC Beijnen JH Li G van Tellingen O. ABCB1 and ABCG2 restrict the brain penetration of a panel of novel EZH2-Inhibitors. Int J Cancer (2015) 137:2007–18. 10.1002/ijc.29566 25868794

[7] Roussel MF Stripay JL. Epigenetic drivers in pediatric medulloblastoma. Cerebellum (2018) 17:28–36. 10.1007/s12311-017-0899-9 29178021 PMC5807456

[8] Yi J Wu J. Epigenetic regulation in medulloblastoma. Mol Cell Neurosci. (2018) 87:65–76. 10.1016/j.mcn.2017.09.003 29269116 PMC5828953

[9] Zwergel C Valente S Jacob C Mai A. Emerging approaches for histone deacetylase inhibitor drug discovery. Exp Opin Drug Discov. (2015) 10:599–613. 10.1517/17460441.2015.1038236 25895649

[10] Ecker J Oehme I Mazitschek R Korshunov A Kool M Hielscher T. Targeting class I histone deacetylase 2 in MYC amplified group 3 medulloblastoma. Acta Neuropathol Commun. (2015) 3:22. 10.1186/s40478-015-0201-7 25853389 PMC4382927

[11] Li X-N Shu Q Su JM-F Perlaky L Blaney SM Lau CC. Valproic acid induces growth arrest, apoptosis, and senescence in medulloblastomas by increasing histone hyperacetylation and regulating expression of p21Cip1, CDK4, and CMYC. Mol Cancer Ther. (2005) 4:1912–22. 10.1158/1535-7163.MCT-05-0184 16373706

[12] Shu Q Antalffy B Su JMF Adesina A Ou C-N Pietsch T. Valproic acid prolongs survival time of severe combined immunodeficient mice bearing intracerebellar orthotopic medulloblastoma xenografts. Clin Cancer Res. (2006) 12:4687–94. 10.1158/1078-0432.CCR-05-2849 16899619

[13] Yuan J Llamas Luceno N Sander B Golas MM. Synergistic anti-cancer effects of epigenetic drugs on medulloblastoma cells. Cell Oncol. (2017) 40:263–79. 10.1007/s13402-017-0319-7 28429280 PMC13001549

[14] Phi JH Choi SA Kwak PA Lee JY Wang KC Hwang DW. Panobinostat, a histone deacetylase inhibitor, suppresses leptomeningeal seeding in a medulloblastoma animal model. Oncotarget (2017) 8:56747–57. 10.18632/oncotarget.18132 28915627 PMC5593598

[15] Milde T Lodrini M Savelyeva L Korshunov A Kool M Brueckner LM. HD-MB03 is a novel group 3 medulloblastoma model demonstrating sensitivity to histone deacetylase inhibitor treatment. J Neuro Oncol. (2012) 110:335–48. 10.1007/s11060-012-0978-1 23054560

[16] Canettieri G Di Marcotullio L Greco A Coni S Antonucci L Infante P Histone deacetylase and Cullin3-RENKCTD11 ubiquitin ligase interplay regulates Hedgehog signalling through Gli acetylation. Nat Cell Biol. (2010) 12:132–42. 10.1038/ncb2013 20081843

[17] Lee SJ Krauthauser C Maduskuie V Fawcett PT Olson JM Rajasekaran SA. Curcumin-induced HDAC inhibition and attenuation of medulloblastoma growth *in vitro* and *in vivo*. BMC Cancer (2011) 11:144. 10.1186/1471-2407-11-144 21501498 PMC3090367

[18] Kolbinger FR Koeneke E Ridinger J Heimburg T Muller M Bayer T. The HDAC6/8/10 inhibitor TH34 induces DNA damage-mediated cell death in human high-grade neuroblastoma cell lines. Arch Toxicol. (2018) 92:2649–64. 10.1007/s00204-018-2234-8 29947893 PMC6063332

[19] Rathkopf DE Picus J Hussain A Ellard S Chi KN Nydam T. A phase 2 study of intravenous panobinostat in patients with castration-resistant prostate cancer. Cancer Chemother Pharmacol. (2013) 72:537–44. 10.1007/s00280-013-2224-8 23820963 PMC3970811

[20] Begley CG Ellis LM. Drug development: raise standards for preclinical cancer research. Nature (2012) 483:531–3. 10.1038/483531a 22460880

[21] Smith MA Houghton P. A proposal regarding reporting of *in vitro* testing results. Clin Cancer Res. (2013) 19:2828–33. 10.1158/1078-0432.CCR-13-0043 23580781 PMC3741962

[22] Schiedel M Robaa D Rumpf T Sippl W Jung M. The current state of NAD^+^ -dependent histone deacetylases (sirtuins) as novel therapeutic targets. Med Res Rev. (2018) 38:147–200. 10.1002/med.21436 28094444

[23] Ma J-X Li H Chen X-M Yang X-H Wang Q Wu M-L. Expression patterns and potential roles of SIRT1 in human medulloblastoma cells *in vivo* and *in vitro*. Neuropathology (2013) 33:7–16. 10.1111/j.1440-1789.2012.01318.x 22537175

[24] Tiberi L Bonnefont J van den Ameele J Le Bon S-D Herpoel A Bilheu A. A BCL6/BCOR/SIRT1 complex triggers neurogenesis and suppresses medulloblastoma by repressing sonic hedgehog signaling. Cancer Cell (2014) 26:797–812. 10.1016/j.ccell.2014.10.021 25490446

[25] Wadhwa E Nicolaides T. Bromodomain inhibitor review: bromodomain and extra-terminal family protein inhibitors as a potential new therapy in central nervous system tumors. Cureus (2016) 8:e620. 10.7759/cureus.620 27382528 PMC4917374

[26] Loven J Hoke HA Lin CY Lau A Orlando DA Vakoc CR. Selective inhibition of tumor oncogenes by disruption of super-enhancers. Cell (2013) 153:320–34. 10.1016/j.cell.2013.03.036 23582323 PMC3760967

[27] Tang Y Gholamin S Schubert S Willardson MI Lee A Bandopadhayay P. Epigenetic targeting of Hedgehog pathway transcriptional output through BET bromodomain inhibition. Nat Med. (2014) 20:732–40. 10.1038/nm.3613 24973920 PMC4108909

[28] Henssen A Thor T Odersky A Heukamp L El-Hindy N Beckers A. BET bromodomain protein inhibition is a therapeutic option for medulloblastoma. Oncotarget (2013) 4:2080–95. 10.18632/oncotarget.1534 24231268 PMC3875771

[29] Bandopadhayay P Bergthold G Nguyen B Schubert S Gholamin S Tang Y. BET bromodomain inhibition of MYC-amplified medulloblastoma. Clin Cancer Res. (2014) 20:912–25. 10.1158/1078-0432.CCR-13-2281 24297863 PMC4198154

[30] Venkataraman S Alimova I Fan R Harris P Foreman N Vibhakar R. MicroRNA 128a increases intracellular ROS level by targeting Bmi-1 and inhibits medulloblastoma cancer cell growth by promoting senescence. PLoS ONE (2010) 5:e10748. 10.1371/journal.pone.0010748 20574517 PMC2888574

[31] Long J Li B Rodriguez-Blanco J Pastori C Volmar C-H Wahlestedt C. The BET bromodomain inhibitor I-BET151 acts downstream of smoothened protein to abrogate the growth of hedgehog protein-driven cancers. J Biol Chem. (2014) 289:35494–502. 10.1074/jbc.M114.595348 25355313 PMC4271234

[32] Bolin S Borgenvik A Persson CU Sundstrom A Qi J Bradner JE. Combined BET bromodomain and CDK2 inhibition in MYC-driven medulloblastoma. Oncogene (2018) 37:2850–62. 10.1038/s41388-018-0135-1 29511348 PMC5966365

[33] Alimova I Venkataraman S Harris P Marquez VE Northcott PA Dubuc A. Targeting the enhancer of zeste homologue 2 in medulloblastoma. Int J Cancer (2012) 131:1800–9. 10.1002/ijc.27455 22287205 PMC3375399

[34] Miele E Valente S Alfano V Silvano M Mellini P Borovika D. The histone methyltransferase EZH2 as a druggable target in SHH medulloblastoma cancer stem cells. Oncotarget (2017) 8:68557–70. 10.18632/oncotarget.19782 28978137 PMC5620277

[35] Dobson THW Hatcher RJ Swaminathan J Das CM Shaik S Tao RH. Regulation of USP37 expression by REST-associated G9a-dependent histone methylation. Mol Cancer Res. (2017) 15:1073–84. 10.1158/1541-7786.MCR-16-0424 28483947 PMC5540785

[36] Inui K Zhao Z Yuan J Jayaprakash S Le LTM Drakulic S. Stepwise assembly of functional C-terminal REST/NRSF transcriptional repressor complexes as a drug target. Protein Sci. (2017) 26:997–1011. 10.1002/pro.3142 28218430 PMC5405421

[37] Zwergel C Valente S Mai A. DNA methyltransferases inhibitors from natural sources. Curr Top Med Chem. (2016) 16:680–96. 10.2174/1568026615666150825141505 26303417

[38] Marino A-M Frijhoff J Calero R Baryawno N Oestman A Johnsen JI. Effects of epigenetic modificators in combination with small molecule inhibitors of receptor tyrosine kinases on medulloblastoma growth. Biochem Biophys Res Commun. (2014) 450:1600–5. 10.1016/j.bbrc.2014.07.042 25026552

[39] Andrade AF Borges KS Suazo VK Geron L Correa CA Castro-Gamero AM The DNA methyltransferase inhibitor zebularine exerts antitumor effects and reveals BATF2 as a poor prognostic marker for childhood medulloblastoma. Invest New Drugs (2017) 35:26–36. 10.1007/s10637-016-0401-4 27785591

[40] Valente S Liu Y Schnekenburger M Zwergel C Cosconati S Gros C. Selective non-nucleoside inhibitors of human DNA methyltransferases active in cancer including in cancer stem cells. J Med Chem. (2014) 57:701–13. 10.1021/jm4012627 24387159 PMC3983372

[41] Patties I Kortmann RD Glasow A. Inhibitory effects of epigenetic modulators and differentiation inducers on human medulloblastoma cell lines. J Exp Clin Cancer Res. (2013) 32:27. 10.1186/1756-9966-32-27 23672687 PMC3666942

[42] Patties I Kortmann RD Menzel F Glasow A. Enhanced inhibition of clonogenic survival of human medulloblastoma cells by multimodal treatment with ionizing irradiation, epigenetic modifiers, and differentiation-inducing drugs. J Exp Clin Cancer Res. (2016) 35:94. 10.1186/s13046-016-0376-1 27317342 PMC4912728

[43] Muscal JA Scorsone KA Zhang L Ecsedy JA Berg SL. Additive effects of vorinostat and MLN8237 in pediatric leukemia, medulloblastoma, and neuroblastoma cell lines. Invest New Drugs (2013) 31:39–45. 10.1007/s10637-012-9831-9 22669335 PMC3655801

[44] Zhao J Quan H Xie C Lou L. NL-103, a novel dual-targeted inhibitor of histone deacetylases and hedgehog pathway, effectively overcomes vismodegib resistance conferred by Smo mutations. Pharmacol Res Perspect. (2014) 2:e00043. 10.1002/prp2.43 25505589 PMC4186412

